# Effects of structured exercises on selected psychological domains in individuals with type 2 diabetes mellitus in Southern Ethiopia institution-based study

**DOI:** 10.1186/s13102-022-00574-3

**Published:** 2022-10-12

**Authors:** Muluken Gebeyehu, Kessatie Legesse, Soumitra Mondal, Mahmud Abdulkadir, Zeru Bekelle, Alemayehu Molla

**Affiliations:** 1 Department of Sports Science, College of Natural and Computational Science, Dilla University, Dilla, Ethiopia; 2 Department of Sport Science, College of Natural and Computational Science, Mekelle University, Mekelle, Ethiopia; 3 Departments of Biomedical Science, College of Health Science and Medicine, Mekelle University, Mekelle, Ethiopia; 4 Department of Sports Science, College of Natural and Sports Science, Addis Ababa University, Addis Ababa, Ethiopia; 5 Department of psychiatry, College of Health Science and medicine, Dilla University, Dilla, Ethiopia

**Keywords:** GHQ-12, Psychological wellbeing, Structured exercise, Type 2 diabetes mellitus

## Abstract

**Background:**

Psychological disorders are common among individuals with type 2 diabetes mellitus. The effect of exercise training on psychological disorders like anxiety and depression has not been well studied in Ethiopia. The objective of this study was to assess the effect of structured aerobic and resistance exercises on psychological domains among people with type 2 diabetes mellitus in southern Ethiopia.

**Method:**

We began by approaching 97 type 2 diabetic patients who regularly attend follow-up clinics. The 24 study participants were randomly assigned to one of four groups: aerobic exercise (n = 6), resistance exercise (n = 6), combined aerobic and resistance exercise (n = 6), and control group (n = 6). For each arm, the interventions were a structured workout package that was completed without interrupting usual clinical follow-up. The control groups, on the other hand, were kept as follow-up clinical cases with daily routine work. A paired t-test was performed to compare the means and mean differences of each exercise group’s pretest and posttest. A Tukey’s post hoc test was performed to compare mean differences between groups. The significance level was set at P < 0.05.

**Result:**

A more significant change was obtained in the combined aerobic and resistance exercise on anxiety and depression (p = 0.006), social dysfunction (p = 0.009), loss of confidence (p = 0.012) in particular, and psychological wellbeing (p = 0.010) in general. Resistance exercise alone had a significant impact on social dysfunction (p = 0.010), anxiety and depression (p = 0.017), and psychological well-being (p = 0.012) in general. Changes in social dysfunction (p = 0.010), anxiety and depression (p = 0.030), and psychological well-being (p = 0.018) were also influenced by aerobic exercise. The study found no significant change in depression and anxiety among control groups.

**Conclusion:**

As a result, the combined effect of aerobic and resistance exercise had a greater influence on improving anxiety and depression, social dysfunction, loss of confidence in particular, and psychological well-being in general among patients with type 2 diabetes. In other words, the present evidence proves the betterment of combined aerobic and resistance exercises alone followed by resistance exercise alone and aerobic exercise alone compared with non-exercise groups seems to upgrade psychological wellbeing by setting up three main benefits named as: (1) anti-anxiety and depression effects; (2) anti-social dysfunction effects; and (3) anti-loss of confidence effects. This suggests that patients with diabetes who exercise regularly enhance their psychological well-being.

**Supplementary Information:**

The online version contains supplementary material available at 10.1186/s13102-022-00574-3.

## Introduction

Diabetes mellitus is a collection of metabolic diseases marked by high blood glucose levels caused by the body’s inability to produce and/or utilize insulin [1]. Diabetes will affect over 439 million adults worldwide by 2030, accounting for 7.7% of the global population [2]. Diabetes is predicted to affect around 19.8 million people in Africa. And, in Ethiopia, 1.9 million patients with this non-communicable disease are estimated to exist among Africa’s diabetes population [3]. In this population of Ethiopia, 1.3 million of the adult population had type 2 diabetes mellitus [4]. The urban population’s type 2 diabetes population outnumbers the rural population [5, 6].

Obesity, lack of exercise, stress, and aging are all factors that contribute to type 2 diabetes [5]. Diabetes-related stress develops depression and other psychological problems, and depression is a prevalent concomitant health concern among Ethiopian patients with type 2 diabetes[6]. Furthermore, recent studies show that people with diabetes are more likely to be diagnosed with co-morbid depression and anxiety than their non-diabetic counterparts[7]. Type-2 diabetes has a negative impact on all life domains, particularly on “future,“ “freedom to consume,“ and " confidence“[8]. “Worrying about the future and the prospect of significant problems” was the most commonly stated, followed by “guilt or anxiety during diabetes control”.

To reduce the risk of type 2 diabetes complications, long-term lifestyle adjustments and multi-pharmacological treatment are required. Physical activity and planned exercise are crucial in achieving this goal [9]. And, as part of the continuous medical management of type 2 diabetes, a psychological and social situation assessment should be incorporated to assist in decreasing and/or postponing the comorbidities of depression, anxiety, eating disorders, cognitive impairment, and enhance adherence to the medical regimen [10].

As a result, physical activity has been linked to better mental health and psychological well-being in people with type 2 diabetes [9]. Physical activity and psychological well-being are widely acknowledged to play important roles in personal and public health, with physical activity being widely promoted as a treatment for common, debilitating social and psychological problems [13]. Furthermore, increased levels of endorphins (which improve mood) and norepinephrine regulation (which improves mood and decreases the physiologic symptoms of depression[11], such as feeling rundown, having trouble sleeping, and having trouble concentrating) or social psychological processes such as improved self-esteem, confidence, and improved concentration may facilitate the link between physical exercise (a subset of physical activities) and psychological wellbeing. Regular, moderate-intensity physical exercise interventions, in which the type, intensity, and duration of exercise regimens are tailored to the specific exercisers [12], are particularly important in enhancing health and psychological wellbeing.

The early detection and treatment of psychological problems, particularly in primary health care settings, may help to reduce the harmful outcomes. Screening tests, which have been conducted with original or modified screening instruments or questionnaires [13], are the most common method employed in primary health care centers for diagnosing psychological problems.

We found no comparable studies comparing the independent effects of aerobic exercise, resistance exercise, combined aerobic resistance exercise, and control groups on secondary outcomes such as anxiety and depression, social dysfunction, and loss of confidence in particular and psychological wellbeing in general. On the other hand, Abbas et al., looked into the psychological well-being effects of these workouts on men and women with type 2 diabetes [14]. However, this study looked at the independent outcomes of each group through exercise intervention on anxiety and depression, social dysfunction, and loss of confidence in particular and psychological wellbeing in general.

## Methods

### Subjects

A screening process was established to select participants based on medical records. We found 24 sedentary individuals (16 men and 8 women) with an age range of 40–64. Patients from one to seven years of follow-up type 2 diabetes were recruited by general practitioners of Dilla University Referral Hospital. In addition, the study participants were included when they were following physician check up in the clinic of the endocrinology unit of the hospital; those who would give their consent to participate in aerobic exercise and strength exercise according to the ADA (American Diabetic Associations) and ACSM (American College of Sports Medicine) joint position statement that Dilla University Community Gymnasium (DUCG) was using were included in the study [15]. All 24 included participants with type 2 diabetes completed the study.

### Ethical considerations

The researchers informed the subjects about the study objectives and that their information obtained before pretest and after 16 weeks of exercise training was treated as privileged and confidential. The subjects were also informed that their involvement in the study was voluntary, and they could opt out at any time. There was no financial incentive to attract participants in the study. Both verbal and written instructions on paper were given to confirm that the items were understood (i.e., honorably and without restrictions, the study participants should choose and circle what they believe to have recently existed; this is according to each item question of GHQ-12).

### Study design

A quasi-experimental design was used to evaluate the effects of 4 months of aerobic and resistance exercises on selected psychological domains in individuals with type 2 diabetes mellitus in Dilla, Ethiopia.

### Calculation of sample size

The sample size was determined using a cohort (follow-up) study as a foundation. For the computation of the sample size, we used the following presumptions: The outcome was and the intervention to control ratio was 1:2. (psychological well-being). When we used the adjustment factor, the maximum study population in the follow-up clinic was only 97, making the sample size incredibly tiny. Although this was done since the intervention group assumption was accepted [19] (“and lost to a follow-up rate of 10%”), the adjustment factor was still used. Then we decided to abandon this and use intentional sampling instead (study participants are divided into a control group and an intervention group). Also abandoned was the computation of the statistical sample size due to the study’s inclusion of all qualified participants.

### Tools for data collection

A widely validated shortest form of the questionnaire (GHQ-12) was used [16, 17]. Our study used the translated version of the Amharic (the national language of Ethiopia) language.

The GHQ-12 is used in busy clinical settings and accepted as a screening tool by the international World Health Organization (WHO). The items of the GHQ-12 were selected from a pool of 60 items, including the original questionnaire, GHQ-60. The focus of items is on the inability to carry out normal activities and the appearance of new and distressing symptoms, i.e., depression, anxiety, and observable behaviors [18]. The General Health Questionnaire (GHQ-12) has potential indicators to assess the severity of a psychological disorder that has recently been experienced using a 4-point scale (from 0 to 3). The score was used to mark a total score ranging from 0 to 36, with higher scores signifying worse conditions [19].

There is disagreement among some studies on the factor structure of GHQ-12 [20]. However, the three-factor model (which is formed with factor I—anxiety and depression, factor II—social dysfunction, and factor III—loss of confidence) of GHQ-12, recognized by Graetz in 1991, was a better fit than the one-factor model (and others), and this factor structure was found to be the same for men and women in order to test meaningfully [21].

In our study, this has been evaluated by the effect of aerobic and resistance exercises on selected psychological domains, such as factor I-anxiety and depression, factor II-social dysfunction, and factor III-loss of confidence, independently. And the sum of these factors can measure psychological wellbeing in general among the four allocated groups of type 2 diabetes by using the GHQ-12 questionnaire [22].

### The protocol for exercise intervention

Over the course of the intervention’s four months, walking a mile counted as aerobic exercise. With no more than a day between workouts, the aerobic exercise group engaged in exercises for up to 50 min per day, five days a week, with a moderate intensity set at a rate of felt effort. Free weights, biceps and abs, chest press, shoulder press, leg press, and leg extension were all forms of resistance training. Three times a week on days other than consecutive ones, the resistance exercise group engaged in exercises that lasted for forty minutes. The resistance exercise training load was determined using a percentage of 1RM, which was used in our study to evaluate each participant for each exercise.

The fitness experts at Dilla University Community Gymnasium (DUCG) created the aforementioned resistance and aerobic training routines, which were performed by the combined aerobic and resistance exercise groups for 40 min, four times per week. The control group, on the other hand, was maintained for a period of four months as a regular control (follow-up clinical case residing in the daily routine). Stretching, 10 min intervals of brisk walking, jogging, and stationary cycling served as warm-up exercises throughout each gym session. Each exercise group performed a 5-minute cool-down that included light stretching and waking after the warm-up.

### Statistical analysis

A descriptive statistical analysis was performed to see the normality and homogeneity of some variables. A paired sample t test was used to compare means and the mean differences between the pretest and posttest of groups. To get a significant ANOVA by comparing the changes among groups, we also used Tukey’s post hoc test. Statistical analyses were performed using SPSS version 20 software.

## Results

In this study, we assessed the comparative base line of included variables. Table 1 shows the comparative equivalence of the involved participants on whether they have been influenced by the included variables or not, when they are allocated to the groups of structured exercises. Thus, the study participants’ average baseline age, year of duration of type II diabetes, Glycosylated hemoglobin (HbA1-c), gender( male and female), blood pressure(systolic and diastolic), body weight, and body mass index were assessed in the four allocated groups. The result shows the absence of a significant difference between each group.

**Table 1:** Shows baseline characteristics for homogeneity of groups by their mean

Variable at Base Line	Exercise groups Mean & Standard Deviation(±)				P Value
	**Aerobic**	**Resistance**	**Combined**	**control**	
Age	52 ± 7.3	54.8 ± 5.6	54.8 ± 8.9	53.2 ± 10.4	0.917
Year of duration of type 2 diabetes.	3.3 ± 1.7	3.3 ± 1.5	3.3 ± 2.3	3.3 ± 2.3	1.00
Glycosylated hemoglobin (HbA1-c)	8.3 ± 2.2	8.7 ± 1.5	7.8 ± 1.4	8.8 ± 2.1	0.787
Gender	0.5 ± 0.55	0.5 ± 0.55	0.5 ± 0.55	0.5 ± 0.55	1.00
Blood pressure(Systolic)	133.3 ± 17.5	130 ± 8.9	131.7 ± 9.8	126.7 ± 15.1	0.844
Blood pressure(Diastolic)	81.7 ± 4.1	83.3 ± 5.2	81.7 ± 4.1	75 ± 8.4	0.087
Body weight	71.9 ± 13.8	74.2 ± 10	74.4 ± 13	69.4 ± 4.6	0.843
Body mass index	26.4 ± 4.4	25.6 ± 1.7	24.76 ± 3.3	26.17 ± 4.4	0.866

Notes: P value set at < 0.05

Regarding the effect of aerobic and resistance exercises on selected psychological variables, Table 2 shows the comparative values of pretest and posttest of Factor I (anxiety and depression), Factor II (social dysfunction), and Factor III (loss of confidence) in particular, and psychological wellbeing in general of the four allocated groups. Anxiety and depression, and social dysfunction showed significant changes in the exercised groups, while no significant changes were observed in the control group. Changes in anxiety and depression for both aerobic and resistance exercise groups showed P < 0.05 whereas, the combined aerobic and resistance exercise group showed P < 0.01. Both aerobic and resistance exercises showed P = 0.01 for social dysfunction; however, combined aerobic and resistance exercise showed P < 0.01. Loss of confidence showed a significant reduction (P < 0.05) only in the combined aerobic resistance exercise group. On the other hand, psychological wellbeing (the sum of three factors) showed significant changes in both exercise groups. But no change was observed in the control group. While observing changes in psychological wellbeing in detail, the same change is observed between aerobic exercise and resistance exercise groups (P < 0.05) compared with the combined aerobic and resistance exercise group P = 0.01.

**Table 2:** Comparison of pre exercise intervention and post exercise intervention values of tested psychological domains or variables (Means ± Standard Deviation)

Dimension	Associated Scores (12)	Aerobic exercise group	P Value	Resistance exercise group	P Value	Combined aerobic and resistance exercise group	P Value	Control group	P Value
Factor I (Anxiety & Depression) tested by GHQ 12									
Pre-test		6.5 ± 3.8	0.030	9 ± 3.5	0.017	8.5 ± 4.1	0.006	4 ± 3.7	0.063
Post-test		1.2 ± 0.75		2.5 ± 1.2		1.5 ± 0.8		9.2 ± 3.4	
Factor II ( Social Dysfunction) tested by GHQ 12									
Pre-test		10.8 ± 5.2	0.010	11.5 ± 4.3	0.010	12.2 ± 6.4	0.009	5.3 ± 6.9	0.056
Post- test		0.83 ± 1.3		2 ± 2.2		0.8 ± 1.3		14 ± 3.5	
Factor III (Loss of Confidence) tested by GHQ 12									
Pre-test		2.7 ± 2.3	0.052	3.3 ± 2.2	0.051	4 ± 2.1	0.012	1.8 ± 1.9	0.062
Post-test		0.3 ± 0.8		0.5 ± 0.8		0.5 ± 0.8		4.5 ± 1.4	
Sum of Factor I, Factor II and Factor III (Psychological Wellbeing) tested by GHQ 12									
Pre-test		20 ± 11.2	0.018	23.8 ± 9.3	0.012	24 ± 12.8	0.010	11.2 ± 12.4	0.057
Post-Test		2.3 ± 2.7		5 ± 3.9		2 ± 1.3		27.7 ± 8.1	

Note: P value set at < 0.05, GHQ 12 – General Health Questionnaire of item 12, SD = Standard Deviation

The comparison of differences in variables is shown in Table 3. In accordance with this table, all variables showed significant differences in the four allocated groups, except loss of confidence (P = 0.001), anxiety and depression, social dysfunction, and psychological wellbeing variables showed P < 0.001.

**Table 3:** The comparison of differences in psychological domains or variables during four months in the allocated groups of type 2 diabetes mellitus (Mean ± SD)

Psychological domains (Variables)	Aerobic Exercise Group	Resistance Exercise Group	Combined Aerobic and resistance Exercise group	Control Group	P value
Factor I(Anxiety & Depression) tested by GHQ 12	-5.3 ± 4.4	-6.5 ± 4.5	-7.3 ± 3.9	5.2 ± 5.3	0.000
Factor II (Social Dysfunction) tested by GHQ 12	-10 ± 6	-9.5 ± 5.8	-11.7 ± 7	8.7 ± 8.5	0.000
Factor III(Loss of Confidence) tested by GHQ 12	-2.3±-2.3	-2.8 ± 2.7	-3.7 ± 2.3	2.7 ± 2.7	0.001
Sum of factor I Factor II & Factor III(Psychological wellbeing) tested by GHQ 12	-17.7 ± 12.5	-18.8 ± 12.0	-22 ± 13.3	16.5 ± 16.4	0.000

Note: P value set at < 0.05; SD (±) – Standard Deviation, GHQ 12 - General Health Questionnaire of item 12

The result of inter-group comparisons of significant variables in the four allocated groups was performed using Tukey’s post hoc test in Table 4. The results of the exercised groups on variables of anxiety and depression, social dysfunction, loss of confidence in particular, and psychological wellbeing in general showed significant differences as compared with the control group. Here, aerobic exercise and resistance exercise groups had the same results on anxiety and depression and social dysfunction (P < 0.01, and P = 0.001), whereas, combined aerobic resistance exercise group showed P = 0.001 on anxiety and depression, and P < 0.001on social dysfunction variables. On the other hand, both resistance exercise, and combined aerobic resistance exercise on loss of confidence variable showed P < 0.01 while aerobic exercise group was presented with P < 0.05. The result of both resistance exercise, and combined aerobic resistance exercise on the sum of the three variables (psychological wellbeing) was also presented with P = 0.001, whereas the aerobic exercise group showed P < 0.01.

**Table 4:** Inter-group comparisons of significant variables in the four allocated groups results of the Tukey post hoc test for significant ANOVA

Allocated Groups		Factor I (Anxiety & Depression)	Factor II (Social Dysfunction)	Factor III (Loss of Confidence)	Sum of Factor I, Factor II, & Factor III(Psychological well-being)
Aerobic Exercise Group	Resistance Exercise group	0.971	0.999	0.985	0.999
	Combined Aerobic Resistance Exercise Group	0.923	0.987	0.850	0.971
	Control Group	0.004	0.001	0.012	0.002
Resistance Exercise group	Combined Aerobic Resistance Exercise Group	0.998	0.968	0.966	0.991
	Control Group	0.002*	0.001*	0.006*	0.001*
Combined Aerobic Resistance Exercise Group	Control Group	0.001*	0.000*	0.002*	0.001*

Note:*= P value < 0.05

## Discussion

The improvement of the impact of stressors yet to occur is the provision of exercise in which patients may be enhancing management of diabetes and the way empower themselves to maintain it regardless of the various difficulties in their life [23]. Therefore, consistent emphasis should be given to physical activities and regular exercise that include aerobic exercise and resistance exercise as part of a healthful lifestyle [24].

Participation in regular physical activity is accepted by the participant’s belief in the exercise-psychological well-being association [25]. These associations give a role for physical activities, and structured physical exercises that help the individual to reduce depression and anxiety, improve health and psychological well-being, and enhance work and recreation [26]. Because of the opposite relationship between physical exercise and depression, the study results have indicated that 20 to 40% of diabetics affected by depression who exercised revealed a positive effect on their sense of well-being [27].

The main hypothesis of our study was that the effectiveness of structured exercises on the positively changed psychological domains among groups with type 2 diabetes mellitus was measured in their significantly decreased mean scores after the intervention. This justified the above scholarly studies.

As we have used the twelve-item General Health Questionnaire (GHQ-12), our findings confirm the study of Abbas and his co-authors (24) indicating “all three factors, such as anxiety and depression, social dysfunction, and loss of confidence, together improved psychological wellbeing after a pre-posttest mean score difference among patients with type 2 diabetes mellitus.“

Even though improvement in anxiety and depression was followed by social dysfunction and loss of confidence in the findings of Abbas and his co-authors, our results showed that relatively the same improvement was gained in anxiety and depression, followed by social dysfunction and loss of confidence. From these researchers’ findings, each allocated group, such as aerobic exercise, resistance exercise, or combined aerobic and resistance exercises, did not independently signify their effects and changes in improvement compared with control groups, as this study has found. Hence, to the best of our knowledge, no study has been found that compares between exercise groups and control groups on the three psychological domains in particular, and psychological wellbeing in general, using the 12-item GHQ of the Graetz model in patients with type 2 diabetes mellitus.

Therefore, comparative findings from our study suggest that the effect of combined aerobic and resistance exercises was better than resistance exercise alone or aerobic exercise alone on the three psychological domains (anxiety and depression, social dysfunction, and loss of confidence) in particular and psychological wellbeing in general. While combined aerobic and resistance exercise was followed by resistance exercise and aerobic exercise on anxiety and depression and loss of confidence in terms of the effect of exercise intervention, both exercise groups had the greatest effect on the three psychological domains in particular and psychological wellbeing in general compared to control groups.

The study [28] evaluated the effect of exercise on mental health among people with type 2 diabetes mellitus using short form (SF-36) and WBQ-12 (well-being questionnaire) questionnaires and showed that “aerobic exercise training, resistance exercise training, and a combination of both failed to improve patient-reported wellbeing”. This study reported differences compared with our findings, and this might be due to the different measurement tool.

Other studies have used different measuring scales and confirm our findings on the effects of exercise on psychological domains improvement such as anxiety and depression states and psychological wellbeing as it promotes quality of life [25, 29]. Even if the effects of different exercises on the psychological domains were scattered across different findings with different measuring scales, they show improvement. Except in, the [21]. Same measuring tool (GHQ-12) was used and all three factors improved, as we have indicated in the above findings.

### Conclusion

In our study, we can conclude that combined aerobic and resistance exercises alone can be better than resistance exercise alone and aerobic exercise alone for anxiety and depression, social dysfunction, and loss of confidence improvement in particular, and psychological wellbeing in general. In other words, the present evidence proves the betterment of combined aerobic and resistance exercises alone followed by resistance exercise alone and aerobic exercise alone compared with non-exercise groups seems to upgrade psychological wellbeing by setting up three main benefits named as: (1) anti-anxiety and depression effects; (2) anti-social dysfunction effects; and (3) anti-loss of confidence effects. This suggests that patients with diabetes who exercise regularly enhance their psychological well-being. This suggests that patients with diabetes who exercise regularly enhance their psychological well-being.

### The study’s limitations

The study has the following limitations due to the sample size being somewhat small: Although the maximum study population at the follow-up clinic was only 97, the study population was lost due to a follow-up rate of 10%. The sample size was significantly reduced when the correction factor was applied. The 24 people who were eligible for the study and participated in moderate activity were all relatively few, so the computation of the statistical sample size was likewise scrapped.

## Electronic supplementary material

Below is the link to the electronic supplementary material.

Supplementary Material 1

## Data Availability

All raw data included in the manuscript can be accessed from the corresponding via email address “muluken.gebeyehu21@gmail.com” with reasonable request.
