# Supplementary material for: Effects of structured exercises on selected psychological domains in individuals with type 2 diabetes mellitus in Southern Ethiopia institution-based study
Source: BMC Sports Sci Med Rehabil. 2022 Oct 12;14:181. doi: 10.1186/s13102-022-00574-3 (PMC9558398; doi:10.1186/s13102-022-00574-3)
Supplement: Supplementary file 1 — Supplementary Material 1 [file 13102_2022_574_MOESM1_ESM.docx]

| Answer the12-item questions as the following: | | | | |
| --- | --- | --- | --- | --- |
| Question | 0 | 1 | 2 | 3 |
| 1. Been able to concentrate on what you’re doing | Better than usual | Same as usual | Less than usual | Much less than usual |
| 2. Lost much sleep over worry | Not at all | No more than usual | Rather more than usual | Much more than usual |
| 3. Felt you were playing a useful part in things | More so than usual | Same as usual | Less useful than usual | Much less useful |
| 4. Felt capable of making decisions about things | More so than usual | Same as usual | Less useful than usual | Much less useful |
| 5. Felt constantly under strain | Not at all | No more than usual | Rather more than usual | Much more than usual |
| 6. Felt you couldn’t overcome your difficulties | Not at all | No more than usual | Rather more than usual | Much more than usual |
| 7. Been able to enjoy your normal day-to-day activities | More so than usual | Same as usual | Less useful than usual | Much less useful |
| 8. Been able to face up to your problems | More so than usual | Same as usual | Less useful than usual | Much less useful |
| 9. Been feeling unhappy and depressed | Not at all | No more than usual | Rather more than usual | Much more than usual |
| 10. Been losing confidence in yourself | Not at all | No more than usual | Rather more than usual | Much more than usual |
| 11. Been thinking of yourself as a worthless person | Not at all | No more than usual | Rather more than usual | Much more than usual |
| 12. Been feeling reasonably happy, all things considered | More so than usual | About the same as usual | Less so than usual | Much less than usual |

**Questions and choices for response of the 12-item General Health Questionnaire (GHQ)**

ጥያቄዎችና ምርጫዎች ያሉት በጥናቱ ተሳታፊዎች የሚመለስ 12-ዝርዝር ጉዳይ ለዉ አጠቃላይ የጠና መጠይቅ

| ጥያቄ | | 0 | 1 | 2 | 3 |
| --- | --- | --- | --- | --- | --- |
| 1 | ለምትሰራዉ ስራ አተኩሮ  የመቆየት ጥረት እያደረግህ ነበርህ (ሽ)ወይ | ከተለመደዉ የተሻለ ነዉ | ከተለመደዉ ጋር ተመሳሳይ ነዉ | ከተለመደዉ ያነሰ ነዉ | ከተለመደዉ እጅግ ያነሰ ነዉ |
| 2 | በጭንቀት ምክንያት ብዙ እንቅልፍ አጥተሀል(ሻል) | በጭራሽ | ከተለመደዉ አይበልጥም | ከተለመደዉ ይበልጣል | ከተለመደዉ እጅግ የበለጠ ነዉ |
| 3 | ጠቃሚ ክፈለ ነገሮችን እየሰራህ(ሽ) እንደነበር ተሰምቶሀል(ሻል) | ከተለመደዉ የተሻለ ነዉ | ከተለመደዉ ጋር ተመሳሳይ ነዉ | ከተለመደዉ ያነሰ ነዉ | ከተለመደዉ እጅግ ያነሰ ነዉ |
| 4 | ለነገሮች ዉሳኔመስጠት እንደምትችል ተሰምቶሃል(ሻል) | ከተለመደዉ የተሻለ ነዉ | ከተለመደዉ ጋር ተመሳሳይ ነዉ | ከተለመደዉ ያነሰ ነዉ | ከተለመደዉ እጅግ ያነሰ ነዉ |
| 5 | ዘዉትር በዉጥረት ዉስጥ እንደነበርህ(ሽ) ተሰምቶሀል(ሻል) | በጭራሽ | ከተለመደዉ አይበልጥም | ከተለመደዉ ይበልጣል | ከተለመደዉ እጅግ የበለጠ ነዉ |
| 6 | አስቸጋሪ ነገሮችን መዎጣት እንደማትችል(ይ) ተሰምቶሀል(ሻል) | በጭራሽ | ከተለመደዉ አይበልጥም | ከተለመደዉ ይበልጣል | ከተለመደዉ እጅግ የበለጠ ነዉ |
| 7 | ጠናማ በሆኑ የእለት ተእለት እንቅስቃሴዎችህ(ሽ) ላይ ደስተኛ ለመሆን እየጣርህ(ሽ) ነበረ ወይ | ከተለመደዉ የተሻለ ነዉ | ከተለመደዉ ጋር ተመሳሳይ ነዉ | ከተለመደዉ ያነሰ ነዉ | ከተለመደዉ እጅግ ያነሰ ነዉ |
| 8 | ችግሮችህን (ሽን) ለመጋፈጥ እየጣርህ(ሽ) ነበረ ወይ | ከተለመደዉ የተሻለ ነዉ | ከተለመደዉ ጋር ተመሳሳይ ነዉ | ከተለመደዉ ያነሰ ነዉ | ከተለመደዉ እጅግ ያነሰ ነዉ |
| 9 | ያለመደሰት እና ድብርት ስሜት እተሰማህ እየጣርህ(ሽ) ነበረ ወይ | በጭራሽ | ከተለመደዉ አይበልጥም | ከተለመደዉ ይበልጣል | ከተለመደዉ እጅግ የበለጠ ነዉ |
| 10 | በራስ የመተማመን ችሎታ እያጣህ(ሽ) እየጣርህ(ሽ) ነበረ ወይ | በጭራሽ | ከተለመደዉ አይበልጥም | ከተለመደዉ ይበልጣል | ከተለመደዉ እጅግ የበለጠ ነዉ |
| 11 | እራስህን (ሽን) ዋጋ እንደሌለዉ ሰዉ በማሰብ ላይ እየጣርህ(ሽ) ነበረ ወይ | በጭራሽ | ከተለመደዉ አይበልጥም | ከተለመደዉ ይበልጣል | ከተለመደዉ እጅግ የበለጠ ነዉ |
| 12 | ሁሉንም ነገሮች ግምት ዉስጥ አስገብተህ(ሽ) ምክንያታዊ የሆነ የደስታ ስሜት እየተሰማህ(ሽ) ነበረ ወይ | ከተለመደዉ የተሻለ ነዉ | ከተለመደዉ ጋር ተመሳሳይነት አለዉ | ከተለመደዉ በጣም ያነሰ ነዉ | ከተለመደዉ እጅግ ያነሰ ነዉ |
